# A Redox–Lipid Transcriptional State Is Associated with High Aflatoxin Biosynthetic Activity in *Aspergillus flavus*

**DOI:** 10.3390/metabo16090652

**Published:** 2026-09-05

**Authors:** Yirui Chen, Hongxin Gui, Kai Ma, Ruochen Cao, Zhijian Zhang, Mengyang Wang, Rongrong Yang

**Affiliations:** 1 School of Public Health and Health Sciences, Tianjin University of Traditional Chinese Medicine, No. 10 Poyanghu Road, Jinghai District, Tianjin 301617, China; ciandata@163.com (Y.C.); ghostcixi@163.com (H.G.); imnotcrc@163.com (R.C.); zhangzhijianzzz@163.com (Z.Z.); 2 Department of Electronics, Information and Bioengineering (DEIB), Politecnico di Milano, Piazza Leonardo da Vinci 32, 20133 Milan, Italy; 3 Haihe Laboratory of Modern Chinese Medicine, Tianjin University of Traditional Chinese Medicine, No. 10 Poyanghu Road, Jinghai District, Tianjin 301617, China; yichongma@126.com; 4 Department of Neurobiology, Care Sciences and Society, Karolinska Institutet, Tomtebodavägen 18A, Floor 10, SE-171 65 Solna, Sweden

**Keywords:** Aflatoxin B1, *Aspergillus flavus*, foodomics, oxidative stress, lipid metabolism, food matrix, transcriptomics

## Abstract

**Highlights:**

**What are the main findings?**

- Eight public source studies were harmonized into 18 analytical datasets (247 samples) spanning defined-medium, maize, and peanut systems.
- ABAS agreed descriptively with matched AFB1 measurements; separate models identified a non-monotonic OSAS–ABAS association and a positive OSAS × LMRS interaction.

**What are the implications of the main findings?**

- The coordinated redox–lipid state provides an associative framework for prioritizing candidate pathways and nodes across food-relevant conditions.
- Direct toxin, redox, lipid, flux, temporal, and perturbation studies are needed to test causality and reproducibility.

**Abstract:**

**Background/Objectives:** Aflatoxin B1 (AFB1) contamination of maize and peanut is influenced by fungal responses to environmental and food-matrix cues, but the transcriptional coordination of redox adaptation, lipid metabolism, and aflatoxin biosynthesis remains unresolved. This study examined whether an expression-defined redox–lipid state is associated with high relative aflatoxin biosynthetic activity in *Aspergillus flavus*. **Methods:** A cross-dataset secondary analysis integrated 18 analytical datasets (247 samples) derived from eight public source records spanning defined-medium, maize, and peanut systems. Harmonized expression profiles were used to derive an aflatoxin biosynthetic activity score (ABAS), oxidative-stress adaptation score (OSAS), and lipid metabolic reprogramming score (LMRS). ABAS is an expression-derived relative score, not a direct measure of biosynthetic flux or accumulated toxin. Dataset-aware mixed-effects models evaluated the OSAS–ABAS association and the linear OSAS × LMRS interaction. **Results:** ABAS correlated with matched AFB1 measurements in 54 samples (Pearson r=0.734, $p=2.70\times 10^{-10}$). The quadratic OSAS term was negative (β=−0.434, 95% CI −0.502 to −0.366; p<0.001), with maximum predicted ABAS near 0.81 SD on the pooled within-dataset-standardized OSAS scale. Separately, the linear OSAS × LMRS interaction was positive (β=0.284, 95% CI 0.198–0.370; p<0.001), and the high-OSAS/high-LMRS quadrant had the greatest adjusted mean ABAS (0.789, 95% CI 0.661–0.917). Candidate prioritization recovered established regulators and nominated redox- and lipid-associated nodes. **Conclusions:** The findings identify an associative transcriptional signature across heterogeneous food-relevant conditions and provide focused hypotheses for prospective validation using direct toxin, redox, lipid, flux, and functional measurements.

## 1. Introduction

Aflatoxins are polyketide-derived mycotoxins that compromise the safety of maize, peanut, tree nuts, spices, and animal feeds. Aflatoxin B1 (AFB1), the most potent and prevalent congener, is genotoxic and hepatocarcinogenic, while contamination also causes substantial crop loss and trade disruption. A recent broad synthesis identifies aflatoxigenic *Aspergillus* among the major fungal threats to the global food chain [1]. The principal producer, *Aspergillus flavus*, is metabolically versatile and can colonize crops before harvest and during storage. Temperature, water activity, substrate composition, host genotype, and microbial competition collectively shape fungal growth and toxin accumulation. Climate-driven shifts in mycotoxin risk and the difficulty of breeding durable resistance in maize and peanut further increase the need for mechanistically informed prevention [2,3,4]. Because toxin abundance is an endpoint of fungal adaptation rather than a constitutive property, understanding the coordinated cellular state that permits aflatoxin biosynthesis is central to food-safety control.

The aflatoxin gene cluster contains pathway regulators and structural enzymes whose coordinated expression is embedded in global developmental, epigenetic, and post-translational control systems [5,6,7]. AflR and AflS regulate cluster output, whereas the velvet complex and LaeA connect secondary metabolism to development and environmental perception. Oxidative status is another important layer of regulation. Recent transcriptomic, knockout, and ChIP-seq studies link aflatoxin production to redox genes, Atf-family transcription factors, catalase control, and the SntB antioxidant axis [8,9,10,11,12]. The relationship is unlikely to be strictly monotonic: moderate ROS can act as a signal, whereas excessive oxidative damage constrains growth and biosynthetic capacity. Consistent with this dual role, estragole and curcumin suppress *A. flavus* through perturbation of ROS homeostasis rather than through a uniform antioxidant effect [13,14].

Lipid metabolism could connect oxidative adaptation with aflatoxin biosynthesis through both substrate supply and signaling. Peroxisomal β-oxidation contributes acetyl-CoA, the carbon precursor of polyketide biosynthesis; fatty-acid desaturation alters membrane and redox properties; and oxylipins transmit intra- and inter-organismal signals [15]. Recent metabolomics and lipidomics directly associate temperature-dependent lipid desaturation with aflatoxin production, while functional studies implicate acetyl-CoA carboxylase, FoxA-dependent β-oxidation, and broader carbon-metabolic rewiring in toxin output [16,17,18,19]. Lipid droplets and phospholipid remodeling may additionally coordinate storage, trafficking, and stress adaptation. However, individual studies typically focus on one strain, perturbation, or matrix, making it difficult to distinguish reproducible coupling from dataset-specific responses.

Public resources now span diverse *A. flavus* strains, crop interactions, regulatory perturbations, and gene-expression profiles. AFED provides a recent structured entry point for expression data, and meta-analysis of maize QTL and expression studies illustrates the value of cross-study evidence synthesis [20,21]. Food-matrix sensing is also moving toward sample-level phenotyping, including whole-cell biosensor prediction of AFB1 in moldy maize and peanut kernels [22]. The specific knowledge gap is whether redox adaptation and lipid metabolic reprogramming form a reproducible, joint transcriptional signature of aflatoxin-cluster activity across heterogeneous strains, perturbations, and food matrices. The focus on *A. flavus* reflects its central role in maize and peanut contamination, while the selected lipid pathways represent routes with established links to polyketide precursor supply, membrane adaptation, oxylipin signaling, and cellular lipid storage. The study therefore tested the hypothesis that higher relative aflatoxin biosynthetic activity is associated with coordinated redox adaptation and lipid metabolic reprogramming. Expression-derived scores representing aflatoxin biosynthetic activity (ABAS), oxidative-stress adaptation (OSAS), and lipid metabolic reprogramming (LMRS) were constructed; the shape of the OSAS–ABAS association and the OSAS × LMRS interaction were evaluated; and regulatory and metabolic nodes associated with the high-ABAS state were prioritized.

## 2. Materials and Methods

### 2.1. Study Design and Data Sources

This study was a cross-dataset secondary analysis of publicly available *Aspergillus flavus* transcriptomic data. Candidate datasets were identified through the Aspergillus flavus Expression Database (AFED), and their original sequencing records and experimental metadata were verified using the NCBI Sequence Read Archive, Gene Expression Omnibus, BioProject, USDA Ag Data Commons, and the associated publications [20]. The public source records included SRP034649, PRJNA678857, GSE101899, GSE40202, SRP057550, USDA Ag Data Commons records 25301068 and 25080164, and PRJNA238212. The collection was hypothesis-directed to represent environmental redox stress, food-matrix conditions, lipid-related perturbations, and regulatory or inhibitor perturbations. Eligibility required annotated *A. flavus* samples, identifiable experimental conditions, usable expression data, and sufficient information on strain, growth substrate or food matrix, treatment, sampling, and biological replication. The included environmental contrasts covered oxidative stress, temperature, and water activity; food-matrix contrasts covered defined medium, maize, and peanut systems; lipid-related contrasts covered fatty-acid or oxylipin exposure; and perturbation contrasts covered genetic manipulation and natural-inhibitor treatment.

Distinct experimental contrasts, strains, food matrices, treatments, and sampling conditions within these public source studies were treated as separate analytical datasets. After dataset screening, experimental-condition stratification, and metadata harmonization, 18 analytical datasets comprising 247 samples were included. These units represent analytical groupings derived from eight public source studies and retain their corresponding laboratory, sequencing-batch, and biological contexts. Nine analytical datasets represented defined-medium experiments, five represented maize-associated systems, and four represented peanut-associated systems, contributing 126, 65, and 56 samples, respectively. The public source studies were assigned SRC01–SRC08 in Supplementary Table S1, and the analytical groupings were assigned DS01–DS18 in Supplementary Table S2. Dataset selection, metadata harmonization, expression-data integration, score construction, statistical analyses, and visualization were performed by the authors.

The supplementary source-data package contains the harmonized metadata, author-derived expression scores, model summaries, and figure-source tables generated through these processing steps.

### 2.2. Metadata Harmonization and Analytical Subsets

Sample-level metadata were extracted from the public datasets and harmonized according to fungal strain, experimental condition, growth matrix, temperature, water activity, treatment type, sampling time, biological replicate, and availability of measured AFB1 concentrations. Growth environments were categorized as defined medium, maize, or peanut. Experimental conditions were classified as oxidative stress, temperature exposure, water-activity variation, lipid exposure, genetic perturbation, natural-inhibitor treatment, or food-matrix comparison. The eligible public records contributed multiple strain backgrounds, supporting evaluation across varied biological contexts. Seven analytical datasets contained time-series labels. The source metadata explicitly identify an infection time course in SRC03 (0–144 h, 14 collection times, and eight biological replicates) and three treatment time points in SRC07 (24, 48, and 72 h, with three biological replicates per treatment–time combination). These records do not provide a stable identifier demonstrating that the same biological unit was repeatedly measured across time; the remaining time-labelled groupings likewise lacked a consistent repeated-unit identifier. Time was therefore harmonized as an experimental-condition descriptor rather than modeled as a longitudinal repeated-measures factor. The potential within-series dependence is consequently an unresolved limitation of the present models. The primary integrated analysis included 247 samples. Validation of ABAS included 54 samples with matched transcriptomic and AFB1 measurements. The redox–lipid interaction analysis included 235 samples with complete ABAS, OSAS, and LMRS values; 12 samples with incomplete values for at least one composite score were excluded from that analysis without imputation.

### 2.3. Expression Processing and Quality Control

Transcriptomic expression data and sample annotations were collected from the included public datasets and processed using a unified analytical framework. Gene identifiers were harmonized to consistent *A. flavus* gene symbols before cross-dataset integration. Each expression matrix was retained on its normalized dataset-specific scale. For gene *g* in sample *i* from dataset *d*, expression was standardized across the samples in dataset *d* as

(1) $$Z_{igd}=\frac{E_{igd}-{\bar{E}}_{g,d}}{\mathrm{SD}(E_{g,d})},$$

where $E_{igd}$ is the normalized expression value, and ${\bar{E}}_{g,d}$ and $\mathrm{SD}(E_{g,d})$ are the mean and standard deviation of gene *g* within dataset *d*. Dataset-wise standardization retained within-dataset biological variation while limiting differences in measurement scale among source datasets. Quality control included verification of sample identifiers, biological-group annotations, gene identifiers, missing values, expression-value distributions, and consistency between metadata and expression matrices. Samples lacking information required for a specific analysis were excluded only from that analysis, and missing composite-score values were not imputed.

### 2.4. Aflatoxin Biosynthetic Activity Score

ABAS was constructed from seven representative genes in the aflatoxin biosynthetic cluster: the regulators *aflR* and *aflS*, and the structural genes *aflC*, *aflD*, *aflM*, *aflP*, and *aflQ*. For sample *i* and the K=7 genes, the score was

(2) $${\mathrm{ABAS}}_{i}=\frac{1}{K}\sum_{g=1}^{K}Z_{\mathrm{dataset}}\left(E_{ig}\right),$$

where $E_{ig}$ denotes the expression value of gene *g* in sample *i*, and $Z_{\mathrm{dataset}}\left(E_{ig}\right)$ denotes its standardization within the corresponding dataset. ABAS therefore represents relative, within-dataset transcriptional activation of selected aflatoxin-cluster genes. In this article, “high aflatoxin biosynthetic activity” refers operationally to a higher ABAS within an analytical dataset, whereas biosynthetic flux and accumulated AFB1 are treated as distinct biochemical outcomes. The biological concordance of ABAS was evaluated descriptively in 54 samples with matched AFB1 measurements using Pearson correlation and ordinary least-squares regression.

AFB1 values were retained on the harmonized μg kg^−1^ scale available in the source-data table. The public source studies could differ in matrix, extraction procedure, analytical platform, recovery, normalization basis, sampling time, and limit of detection. No cross-platform recovery correction or analytical-method recalibration was possible from the retained public records; accordingly, the pooled ABAS–AFB1 analysis was interpreted as descriptive concordance across the available matched observations rather than as a calibrated within-study validation. Because *aflR* and *aflS* contributed directly to ABAS construction, their subsequent associations with ABAS were interpreted as internal positive-control evidence rather than independent validation.

### 2.5. Oxidative-Stress Adaptation and Lipid-Reprogramming Scores

Oxidative-stress adaptation was characterized using six predefined functional modules representing ROS sensing, catalase/SOD defense, glutathione metabolism, thioredoxin metabolism, NADPH regeneration, and alternative respiration. These modules were selected to cover sensing, enzymatic ROS removal, thiol-buffering systems, reducing-power regeneration, and respiratory adaptation, which together represent major transcriptional components of fungal oxidative-stress response. Lipid metabolic reprogramming was characterized using six modules representing peroxisomal β-oxidation, fatty-acid desaturation, oxylipin biosynthesis, phospholipid remodeling, acetyl-CoA generation, and lipid-droplet dynamics. These pathways were selected because they encompass precursor supply for polyketide biosynthesis, membrane adaptation, lipid-derived signaling, and lipid storage or mobilization. For module *m* containing $G_{m}$ genes, the sample-level module score was calculated as

(3) $${\mathrm{Module}}_{im}=\frac{1}{G_{m}}\sum_{g=1}^{G_{m}}Z_{igd}.$$

OSAS and LMRS were calculated as the arithmetic means of their six oxidative-stress and lipid-metabolism module scores, respectively. Module definitions were established from functional annotations related to oxidative-stress adaptation, lipid metabolism, and aflatoxin biosynthesis. Higher OSAS and LMRS values indicated stronger relative activation of the corresponding transcriptional programs. The composite scores were evaluated at the module level to preserve a consistent functional scale across datasets.

The retained supplementary source package contains the module-level score tables and functional module labels, but not the original gene-level membership table used during source-data processing. Consequently, the complete gene lists, gene-to-module assignments, and possible gene overlap among modules cannot be independently reconstructed from the current release. No gene was intentionally counted across modules at the composite-score level, but overlap was not formally assessed after the gene-level mapping was discarded. This limitation prevents a module-removal sensitivity analysis for OSAS and LMRS in the present revision and is acknowledged explicitly below.

### 2.6. Nonlinear and Interaction Analyses

The OSAS–ABAS association was examined using a quadratic linear mixed-effects model with a dataset-specific random intercept and two-sided 95% confidence intervals,

(4) $${\mathrm{ABAS}}_{ij}=\beta_{0}+\beta_{1}{\mathrm{OSAS}}_{ij}+\beta_{2}{\mathrm{OSAS}}_{ij}^{2}+u_{j}+\varepsilon_{ij},$$

where *i* indexes samples, *j* indexes analytical datasets, and $u_{j}$ is the dataset-specific random intercept. Evidence for nonlinearity was assessed from the coefficient and two-sided *P* value of the quadratic term. Predicted ABAS values and confidence intervals were calculated across the observed OSAS range. The turning point was calculated as $-\beta_{1}/\left(2\beta_{2}\right)$, and its 95% confidence interval was estimated by dataset-level bootstrap resampling. Because OSAS was standardized within each analytical dataset, this turning point is interpreted as a feature of the pooled standardized data. Dataset-specific linear OSAS estimates were combined using random-effects meta-analysis; $I^{2}$, $\tau^{2}$, and the 95% prediction interval quantified between-dataset heterogeneity.

The joint association of OSAS and LMRS with ABAS was evaluated using a linear mixed-effects interaction model with a dataset-specific random intercept,

(5) $$\begin{array}{cc} {\mathrm{ABAS}}_{ij}= & \beta_{0}+\beta_{1}{\mathrm{OSAS}}_{ij}+\beta_{2}{\mathrm{LMRS}}_{ij}\\ \; & +\beta_{3}\left({\mathrm{OSAS}}_{ij}\times {\mathrm{LMRS}}_{ij}\right)+u_{j}+\varepsilon_{ij}. \end{array}$$

The interaction coefficient $\beta_{3}$ was the primary estimand. Median-split quadrants were used only as an interpretive summary. Cross-dataset robustness was assessed with dataset-specific interaction estimates, random-effects meta-analysis, and 18 leave-one-analytical-dataset-out refits. The interaction model was also fitted separately in defined-medium, maize, and peanut strata; heterogeneity among matrix-specific interaction estimates was evaluated with a two-sided test. These analyses defined robustness at the analytical-dataset and food-matrix levels.

The public source records were identified as SRC01–SRC08, but the retained sample-level analysis tables do not preserve a complete mapping from each observation to its source-study identifier. A leave-one-source-study-out analysis was therefore not feasible from the available release. The reported leave-one-out results should be interpreted strictly as leave-one-analytical-dataset-out sensitivity analyses and not as evidence of independence across the eight source studies.

### 2.7. Module Contrasts and Candidate Prioritization

Within each dataset, samples with ABAS values above the dataset-specific median were classified as AF-high, whereas samples at or below the median were classified as AF-low. These labels served as descriptive, within-dataset categories for visualization and module contrasts, while the primary OSAS and LMRS models used continuous scores. Standardized module differences were reported for each module. Candidate convergence nodes were ranked using meta-log_2_ fold change (0–2 points), ABAS correlation (0–2), module membership (*k*ME; 0–2), motif/ChIP evidence (0–1), perturbation support (0–2), and food-matrix support (0–1), yielding an integrated 0–10 evidence score. The quantitative components were assigned according to prespecified thresholds in the candidate evidence table. Because meta-log_2_ fold change, ABAS correlation, and module membership summarize related transcriptomic information, the weighting is heuristic. The score therefore serves as a transparent prioritization aid. Network panels used the author-generated evidence edge list; weighted connectivity was calculated as the sum of incident edge weights and is presented as an evidence-integration visualization.

### 2.8. Statistical Analysis

Continuous variables were analyzed on their dataset-standardized scales. Pearson correlation evaluated concordance between ABAS and measured AFB1 concentrations. Quadratic and interaction mixed-effects models characterized the OSAS–ABAS and OSAS × LMRS associations, respectively. Dataset-specific effect estimates were combined by random-effects meta-analysis, and leave-one-dataset-out analysis assessed whether the pooled interaction was disproportionately influenced by any analytical dataset. For related module-level tests, *P* values were adjusted with the Benjamini–Hochberg false discovery rate procedure; both two-sided *P* values and FDR-adjusted *q* values are reported. Analyses and visualizations were conducted using Python 3.12.3 with NumPy 2.3.5, pandas 2.3.3, SciPy 1.17.1, Matplotlib 3.10.8, seaborn 0.13.2, and openpyxl 3.1.5. The supplementary source-data package contains the derived model and figure-source tables; the retained release does not contain the original gene-level module map or raw sample-level OSAS/LMRS vectors needed to reconstruct module-removal analyses or an observed-score rug. No additional imputation or outlier deletion was applied.

## 3. Results

### 3.1. Public Transcriptomic Datasets Capture Diverse Food-Related Aflatoxin States

**Figure 1:**
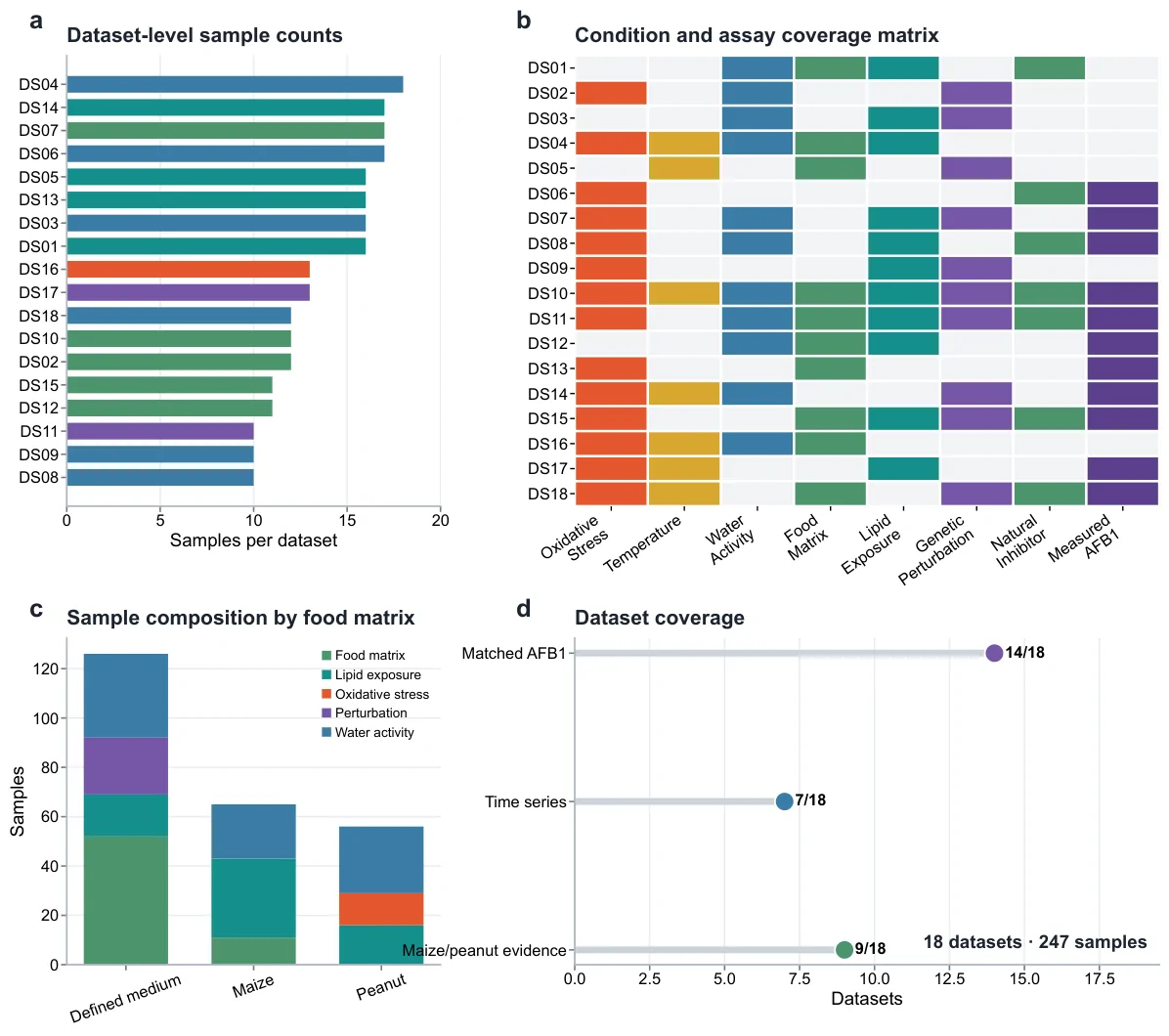
**Composition of the publicly available transcriptomic datasets.** (**a**) Sample count across the 18 analytical datasets, assigned internal identifiers DS01–DS18 for visualization and colored by dominant condition class. (**b**) Coverage of environmental conditions, food matrices, perturbations, and AFB1 measurements. (**c**) Sample composition across defined medium, maize, and peanut systems. (**d**) Availability of matched AFB1 measurements, time-series information, and maize/peanut evidence.

The final analysis comprised 247 samples distributed across defined-medium (126 samples), maize-associated (65), and peanut-associated (56) systems (Figure 1; Table 1). The analytical datasets included 63 NRRL 3357, 59 AF13, 52 CA14, and 73 mixed-isolate samples. Water-activity studies formed the largest condition class (83 samples), followed by lipid exposure (65), food-matrix contrasts (63), perturbation studies (23), and direct oxidative-stress studies (13). Seven analytical datasets were annotated as time series. This breadth enabled evaluation of the redox–lipid hypothesis across multiple strains, matrices, and environmental contexts.

### 3.2. Aflatoxin Biosynthetic Activity Shows a Non-Monotonic Association with OSAS

**Figure 2:**
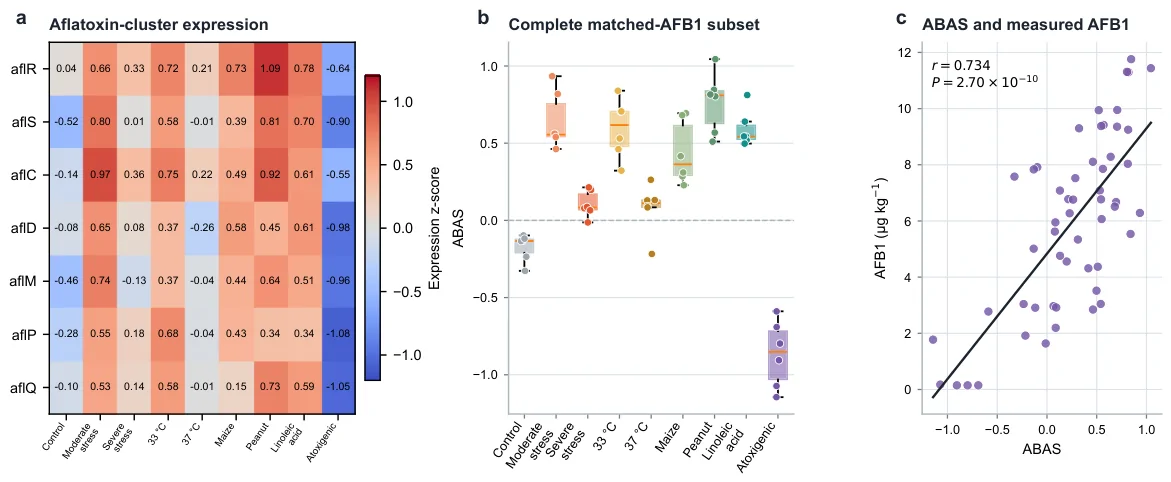
**Aflatoxin biosynthetic activity and cluster behavior.** (**a**) Expression *z*-scores for seven aflatoxin-cluster genes across nine prespecified conditions. (**b**) ABAS distributions with all observations shown (n=6 per condition). (**c**) Association between ABAS and measured AFB1 concentrations (n=54); the line denotes an ordinary least-squares fit. Panels a and b show the complete matched-AFB1 subset without selection by ABAS or AFB1 value.

Seven aflatoxin-cluster genes showed coordinated condition-dependent expression (Figure 2a). In the complete matched-AFB1 subset, mean ABAS was highest under peanut (0.765), followed by moderate oxidative stress (0.644), 33 °C (0.594), linoleic acid (0.593), and maize (0.436). Severe oxidative stress (0.105) and 37 °C (0.081) were lower, whereas the atoxigenic condition had the lowest mean ABAS (−0.867). This descriptive ordering motivated formal evaluation of a non-monotonic association across the standardized OSAS range.

Across the 54 observations with paired toxin values, ABAS correlated with measured AFB1 (Pearson r=0.734, $p=2.70\times 10^{-10}$; Figure 2c). The fitted pooled descriptive slope was 4.46 AFB1 units per ABAS unit. The 54 observations were classified into nine prespecified biological-condition groups with six observations per group; Figure 2 displays the complete matched-AFB1 subset without additional subsampling. This pooled association was interpreted as descriptive concordance across the represented biological conditions and public AFB1 assay contexts.

**Figure 3:**
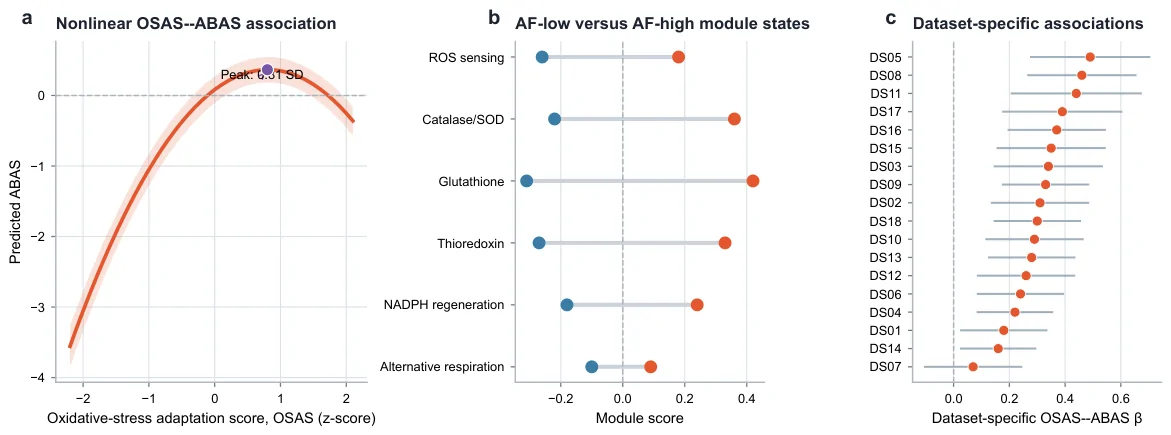
**Oxidative-stress adaptation and nonlinearity.** (**a**) Predicted ABAS across the oxidative-stress adaptation score (OSAS), with the two-sided 95% confidence interval from the quadratic mixed-effects model; the curve is restricted to the observed OSAS range. The retained figure-source table contains the fitted prediction grid but not the raw sample-level OSAS vector, so individual observations are not overlaid. (**b**) Oxidative-module scores in AF-low and AF-high groups defined by dataset-specific ABAS medians; *q* values were obtained with Benjamini–Hochberg correction. (**c**) Dataset-specific OSAS–ABAS estimates with 95% confidence intervals across 18 datasets.

The quadratic mixed-effects model supported a non-monotonic OSAS–ABAS association (Figure 3a). The linear OSAS term was positive (β=0.700, 95% CI 0.610–0.790; p<0.001), whereas the quadratic term was negative (β=−0.434, 95% CI −0.502 to −0.366; p<0.001). Predicted ABAS reached its maximum at an OSAS coordinate of approximately 0.81 SD (95% CI 0.67–0.95) on the pooled within-dataset-standardized scale, where predicted ABAS was 0.366 (95% CI 0.186–0.546), before declining at higher standardized values. This coordinate is interpreted as a fitted feature of the integrated standardized data. Five oxidative modules were higher in the descriptive AF-high than AF-low categories after FDR correction: glutathione (difference 0.73, q<0.001), thioredoxin (0.60, q<0.001), catalase/SOD (0.58, q<0.001), ROS sensing (0.44, q=0.003), and NADPH regeneration (0.42, q=0.004); alternative respiration did not meet the FDR threshold (0.19, q=0.074). Positive analytical-dataset-specific OSAS–ABAS associations were observed in 17 of 18 datasets, with the confidence interval crossing zero only in DS07. The random-effects summary was β=0.289 (95% CI 0.241–0.336; $I^{2}=26.2\%$, $\tau^{2}=0.0027$, 95% prediction interval 0.176–0.402).

### 3.3. High-Aflatoxin States Show Coordinated Lipid Metabolic Reprogramming

**Figure 4:**
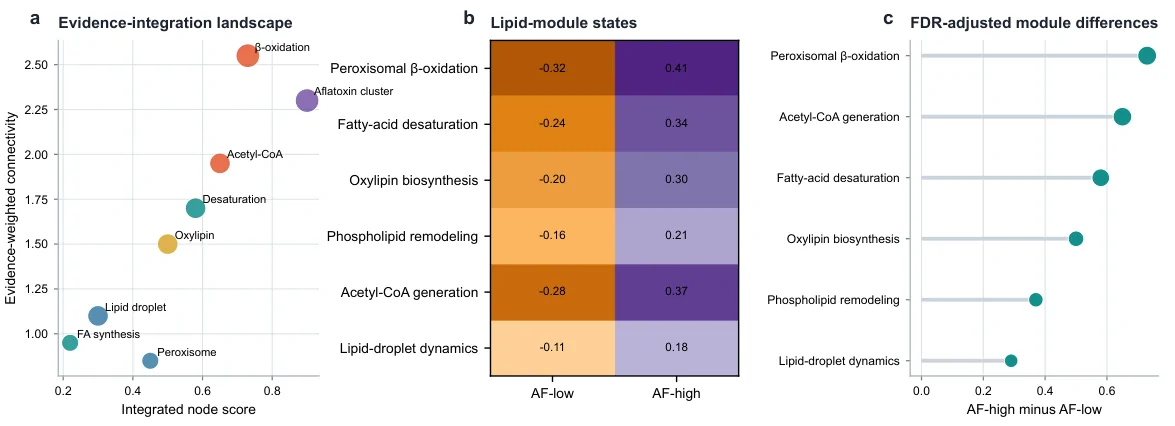
**Lipid metabolic reprogramming.** (**a**) Evidence-list landscape of eight pathway nodes. The *x* axis shows integrated node score, the *y* axis shows weighted connectivity calculated as the sum of incident edge weights, bubble area encodes the number of listed connections, and color denotes pathway class; dashed lines indicate medians. (**b**) Lipid-module scores in AF-low and AF-high groups. (**c**) Standardized module differences, with point area proportional to $-\log_{10}\left(q\right)$.

All six lipid modules were higher in AF-high than AF-low samples after FDR correction (Figure 4b,c). Peroxisomal β-oxidation had the largest standardized difference (0.73, q<0.001), followed by acetyl-CoA generation (0.65, q<0.001), fatty-acid desaturation (0.58, q<0.001), oxylipin biosynthesis (0.50, q=0.003), phospholipid remodeling (0.37, q=0.011), and lipid-droplet dynamics (0.29, q=0.032). The pathway-node display summarized an author-curated evidence list linking lipid processes to aflatoxin-cluster activity; its connectivity values represent evidence-integration quantities.

### 3.4. Redox Adaptation and Lipid Reprogramming Are Jointly Associated with the High-ABAS State

**Figure 5:**
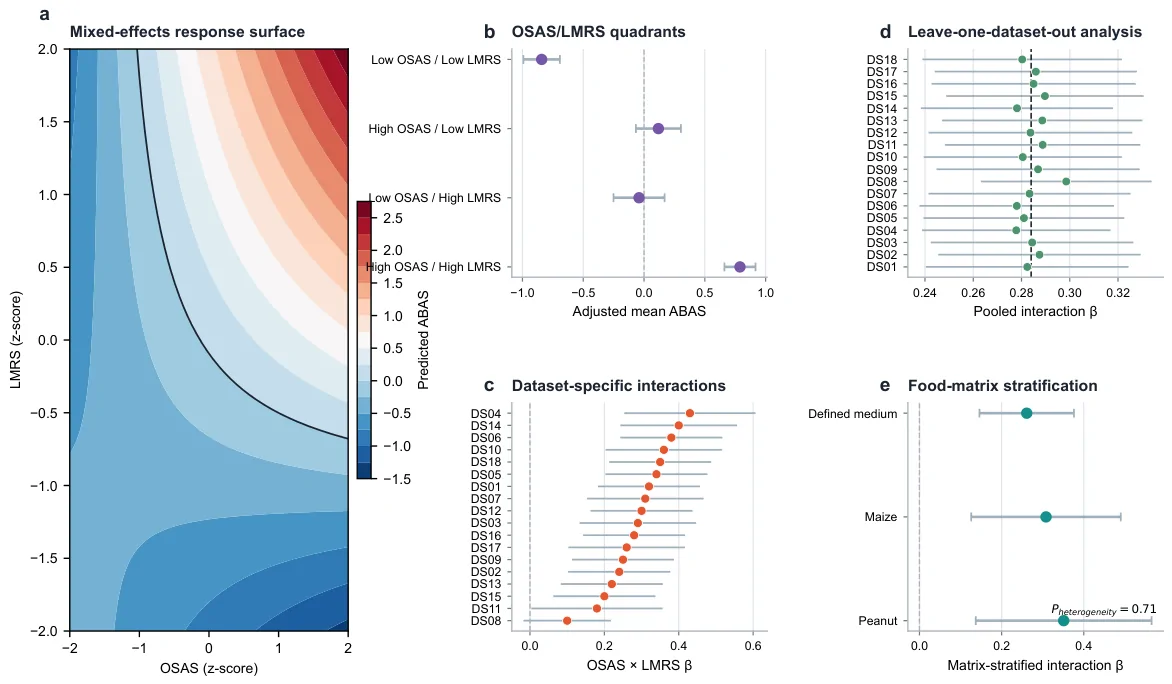
**Redox–lipid convergence.** (**a**) Two-dimensional model-predicted OSAS × LMRS response surface from the primary mixed-effects model; the solid contour indicates predicted ABAS =0. (**b**) Model-adjusted marginal means and 95% confidence intervals in four OSAS/LMRS quadrants, with sample sizes shown for the 235 complete interaction samples. (**c**) Dataset-specific interaction estimates across 18 datasets. (**d**) Leave-one-dataset-out random-effects summaries. (**e**) Matrix-stratified interaction estimates in defined medium, maize, and peanut; $P_{\mathrm{heterogeneity}}=0.71$.

The linear mixed-effects interaction model identified a positive OSAS × LMRS interaction in relation to ABAS (Figure 5a). In the primary model, OSAS (β=0.321, 95% CI 0.259–0.383; p<0.001) and LMRS (β=0.439, 95% CI 0.373–0.505; p<0.001) were each positively associated with ABAS. Their interaction was also positive (β=0.284, 95% CI 0.198–0.370; p<0.001; Table 2). Quadrant summaries were concordant with the continuous linear interaction but remained descriptive. Model-adjusted mean ABAS was −0.842 (95% CI −0.992 to −0.692; n=76) for low OSAS/low LMRS, 0.118 (−0.067 to 0.303; n=43) for high OSAS/low LMRS, −0.041 (−0.251 to 0.169; n=21) for low OSAS/high LMRS, and 0.789 (0.661–0.917; n=95) for high OSAS/high LMRS. The quadratic OSAS model and the linear interaction model address complementary but distinct estimands.

Dataset-specific OSAS × LMRS interaction estimates were positive in 17 of 18 datasets; the confidence interval for DS08 included zero (Figure 5c). The random-effects summary was β=0.284 (95% CI 0.245–0.324; $I^{2}=26.7\%$, $\tau^{2}=0.0020$, 95% prediction interval 0.189–0.380). In leave-one-dataset-out analyses, pooled interaction estimates ranged from 0.278 to 0.299. The interaction was positive in defined-medium (β=0.261, 95% CI 0.146–0.376), maize (β=0.308, 95% CI 0.126–0.490), and peanut (β=0.351, 95% CI 0.137–0.565) strata, with no evidence of between-matrix heterogeneity (p=0.71). The interaction analysis included the 235 samples with complete ABAS, OSAS, and LMRS values; 12 samples with incomplete composite-score values were excluded without imputation. These sensitivity analyses operate at the analytical-dataset level; a source-study-level leave-out analysis was not possible because the retained sample-level release lacks a complete SRC01–SRC08 mapping.

### 3.5. Regulatory and Metabolic Convergence Nodes Connect the Coupling State to Aflatoxin Biosynthesis

**Figure 6:**
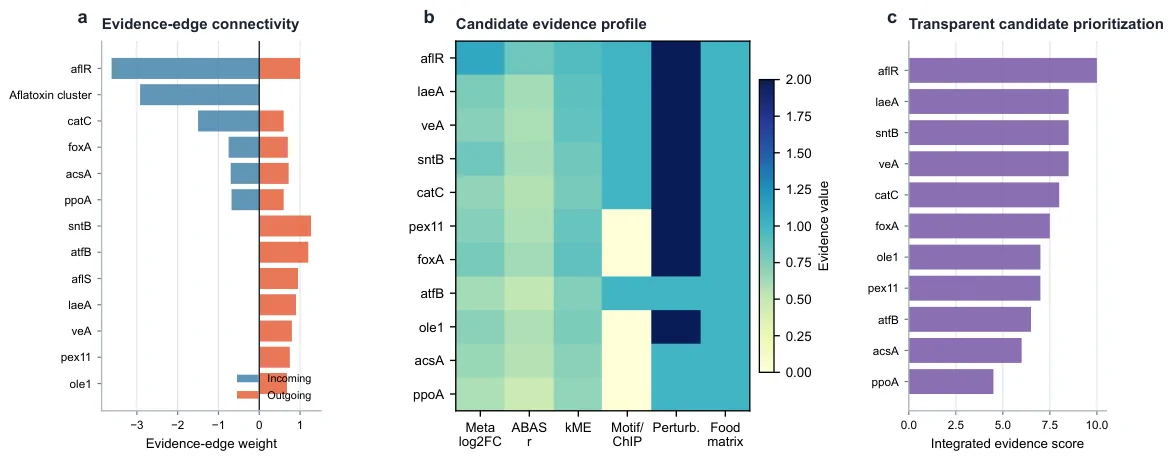
**Candidate regulators and convergence nodes.** (**a**) Diverging weighted-connectivity chart for 13 nodes in the author-generated evidence edge list. Bars to the left show summed incoming edge weight and bars to the right show summed outgoing edge weight; nodes are ordered by total connectivity. (**b**) Evidence profile across six scored components. Color is scaled independently within each nonconstant column and therefore represents relative position rather than a shared unit. (**c**) Integrated evidence scores for 11 candidate genes, sorted for display. The panels visualize evidence integration and do not infer a de novo regulatory network.

The transparent evidence-integration framework prioritized 11 genes spanning established aflatoxin regulators, oxidative-stress responses, and lipid metabolism (Figure 6; Table 3). AflR (10.0) served as the positive-control cluster-regulatory anchor, while LaeA and VeA (each 8.5) provided positive-control global-regulatory anchors. Among genes not used to construct ABAS, SntB (8.5), CatC (8.0), FoxA (7.5), Pex11 (7.0), and Ole1 (7.0) received the strongest combined support from cross-dataset expression, module membership, perturbation evidence, and food-matrix support. These nodes nominate testable links among antioxidant adaptation, peroxisomal function, β-oxidation, desaturation, oxylipin signaling, acetyl-CoA supply, and cluster activation; the integrated scores define relative priorities for follow-up.

## 4. Discussion

### 4.1. Principal Findings

Across heterogeneous food-relevant transcriptomic datasets, relative aflatoxin biosynthetic activity was associated with a coordinated redox–lipid transcriptional state. ABAS showed pooled concordance with paired AFB1 measurements and separated the displayed high-ABAS conditions from the atoxigenic condition. The quadratic mixed-effects model identified a non-monotonic OSAS–ABAS association, whereas a separate linear interaction model identified a positive OSAS × LMRS interaction. Random-effects summaries, leave-one-analytical-dataset-out estimates, and matrix-stratified models were consistent with a positive interaction at the analytical-dataset level. The evidence-integration analysis recovered established secondary-metabolism regulators and identified antioxidant and lipid-metabolic candidates for targeted testing. Together, these findings define an associative transcriptional framework and a focused basis for prospective mechanistic studies.

### 4.2. An Adaptive Redox Window for Aflatoxin Biosynthesis

Recent studies confirm that oxidative state is tightly coupled to *A. flavus* development and aflatoxin production, but they also show strong dependence on stressor, intensity, time, and genetic background [8,9,11]. The observed OSAS pattern provides one transcriptional interpretation. At lower OSAS values, the coordinated stress-adaptation program is less prominent. At moderately elevated values on the pooled standardized scale, ROS sensing is accompanied by catalase/SOD, glutathione, thioredoxin, and NADPH-regeneration programs, a configuration associated with greater predicted ABAS. At higher standardized values, predicted ABAS declines. The ability of estragole and curcumin to inhibit growth and aflatoxin through ROS disequilibrium further illustrates that the direction and magnitude of redox adaptation may influence the phenotype [13,14].

OSAS is a gene-expression score rather than a direct chemical measurement of ROS or oxidative load. The observed peak therefore identifies a relative coordinate in the pooled standardized data rather than a universal physiological threshold. Temporal ordering among redox–lipid transcriptional changes and aflatoxin-cluster activation remains an important question for prospective studies. Joint measurement of ROS, GSH/GSSG, NADPH/NADP^+^, NADH/NAD^+^, antioxidant-enzyme activity, growth, cluster expression, and AFB1 would help distinguish induction, adaptation, feedback, and damage. SntB–CatC regulation and the broad AtfA target landscape show that antioxidant responses and secondary metabolism share upstream control, supporting a bidirectional interpretation [11,12].

### 4.3. Lipid Metabolism as a Substrate and Signaling Bridge

The lipid-module profile suggests two nonexclusive routes by which transcriptional reprogramming could accompany high ABAS. The first is potential substrate supply. Peroxisomal β-oxidation can release acetyl-CoA units and reducing equivalents, while acetyl-CoA is the starting substrate for aflatoxin polyketide synthesis. Coordinated increases in the β-oxidation and acetyl-CoA expression modules could therefore mark a carbon-allocation program associated with secondary metabolism. This interpretation is biologically supported by temperature-resolved lipidomics, acetyl-CoA carboxylase perturbation, and FoxA functional analysis [16,17,18]. The second route is signaling and cellular organization. Fatty-acid desaturation influences membrane properties and susceptibility to peroxidation; oxylipins can mediate fungal development and host–fungus communication; and lipid droplets can buffer or mobilize hydrophobic metabolites [15]. TPS1-linked multi-omic rewiring further shows that carbon metabolism, fatty-acid synthesis, stress adaptation, and cluster transcription can change together [19]. Because LMRS is derived from transcript abundance, direct lipidomics and isotope tracing will be valuable for resolving lipid abundance, synthesis and degradation flux, membrane remodeling, lipid peroxidation, and signaling.

The positive OSAS × LMRS coefficient indicates that the linear association of either score with ABAS depends statistically on the level of the other score in the fitted model. High OSAS with low LMRS produced only modestly positive mean ABAS, whereas high LMRS with low OSAS remained near zero; the descriptive high/high quadrant had the greatest adjusted mean. This pattern is compatible with a permissive state in which antioxidant adaptation sustains cellular function while lipid metabolism supplies precursors and signals. It is interpreted as a statistical interaction, with biochemical coordination remaining a target for direct functional investigation. Stable leave-one-analytical-dataset-out estimates further support consistency across the analytical groupings.

### 4.4. Regulatory Convergence and Testable Candidates

The highest-ranked regulators—LaeA, VeA, and AflR—are expected anchors rather than novel discoveries. Recent reviews synthesize how cluster transcription factors, the velvet system, epigenetic readers, and post-translational modifications coordinate fungal secondary metabolism [5,6,7]. Their recovery supports the face validity of the prioritization scheme. SntB, AtfB, and CatC provide plausible links to oxidative adaptation: the KERS complex and SntB regulate development and antioxidant pathways, while Atf-family factors connect stress sensing to aflatoxin output [9,10,12,23].

Recent perturbation studies also show that this regulatory layer is distributed rather than linear. Histone deacetylase SirE, Set9, SWD1, Afngg1-dependent 2-hydroxyisobutyrylation, and VBS succinylation each affect development and aflatoxin production [24,25,26,27,28]. Environmental and nutrient signals reach the cluster through HacA-mediated unfolded-protein response, m^6^A writers and target transcripts, Fus3–Gal83 signaling, and TOR activity [29,30,31,32,33]. These studies support a convergence framework and position the present candidate network as a guide for targeted experimental validation.

Pex11, FoxA, Ole1, PpoA, and AcsA extend the candidate set into peroxisome proliferation, β-oxidation, desaturation, oxylipin metabolism, and acetyl-CoA generation. These genes are particularly useful for follow-up because they translate a systems-level interaction into perturbable nodes. A minimal validation design would compare control, moderate oxidative stress, severe oxidative stress, linoleic acid, and combined moderate-stress/linoleic-acid conditions. For two or three candidates, gene knockdown or deletion should be paired with AFB1 quantification, growth or biomass, ROS, lipid peroxidation, antioxidant measurements, and expression of structural cluster genes. Rescue or complementation would be required before claiming that a node mediates redox–lipid coupling.

### 4.5. Food-Matrix Relevance

The inclusion of maize and peanut is central to the food-related metabolic context. Both matrices are rich in lipids or lipid-derived signals and differ from defined medium in water availability, nutrient structure, and host defenses. Peanut had the highest mean ABAS among the nine displayed conditions, while maize and linoleic-acid exposure also showed elevated activity. These observations are compatible with matrix-dependent availability of unsaturated fatty acids and oxylipin precursors. Recent crop-focused reviews and meta-analysis emphasize that host resistance, environmental stress, and fungal metabolism jointly determine contamination in maize and peanut [3,4,21]. Their distribution across the integrated datasets likely contributes to heterogeneity.

The positive OSAS × LMRS association was observed in defined-medium, maize, and peanut strata, and the matrix-heterogeneity test was not significant. These results are consistent with a cross-matrix pattern while preserving the possibility that the underlying molecular routes differ among substrates. The framework generates hypotheses for multi-target control strategies involving oxidative adaptation and lipid-metabolic support. Recent proteomic work with eugenol illustrates how a food-compatible intervention can simultaneously disrupt cellular integrity, energy metabolism, acetyl-CoA availability, and aflatoxin biosynthesis [34]. Efficacy, sensory consequences, stability, and matrix compatibility nevertheless require direct testing.

### 4.6. Strengths and Limitations

The study’s main strength is its evaluation of consistency across 18 analytical groupings rather than reliance on a single strain or treatment. The collection spans multiple food matrices and environmental perturbations, while ABAS provides an expression-based proxy related to a toxin phenotype. Dataset random intercepts, analytical-dataset-level random-effects summaries, leave-one-analytical-dataset-out estimates, and matrix-stratified models address part of the heterogeneity in the integrated data. The explicit integration of redox and lipid modules provides a coherent biological hypothesis, and transparent candidate prioritization offers a route to experimental validation.

Interpretation should account for the study’s public-data design. The 18 analytical datasets represent experimental contrasts derived from eight public source records, so effect estimates refer to analytical groupings that span diverse strains, matrices, treatments, assay contexts, and sampling structures. ABAS, OSAS, and LMRS are within-dataset-standardized transcriptional scores, and the pooled association with matched AFB1 values is best viewed as descriptive concordance. The candidate-prioritization score is heuristic because several components summarize related transcriptomic evidence; in addition, *aflR* serves as a positive-control anchor and also contributes to ABAS. The quadratic OSAS model and the linear OSAS × LMRS model address distinct estimands. Source-study-level leave-one-out robustness was not possible because the retained sample-level release lacks a complete SRC01–SRC08 mapping; the analytical-dataset-level leave-one-out results should therefore not be interpreted as source-study independence. Time-series labels were available for seven analytical datasets, but the public metadata did not consistently establish whether observations represented independent cultures or repeated measurements of the same units. Potential within-series dependence remains unresolved. The complete gene-level module map was not retained, so module overlap and module-removal sensitivity could not be evaluated. The observed-score distribution requested for Figure 3a likewise could not be reconstructed from the retained figure-source tables, which contain the fitted curve but not the raw OSAS vector. Accordingly, the findings define an associative, hypothesis-generating framework for prospective validation across controlled biological systems.

## 5. Conclusions

Across diverse food-relevant conditions, relative aflatoxin biosynthetic activity was associated with a redox–lipid transcriptional state characterized by oxidative-stress adaptation and lipid metabolic reprogramming. The quadratic model identified a non-monotonic OSAS–ABAS association on the pooled within-dataset-standardized scale. Separately, the linear interaction model identified a positive OSAS × LMRS association with ABAS. These analytical-dataset-level findings define a potential associative signature and generate focused hypotheses involving antioxidant, peroxisomal, β-oxidation, desaturation, oxylipin, and acetyl-CoA pathways. Prospective studies incorporating direct toxin, redox, lipid, flux, and functional measurements can build on this framework to evaluate temporal relationships, biochemical coordination, and applications in aflatoxin control. 

## Figures and Tables

**Table 1:** Characteristics of the publicly available transcriptomic datasets included in the analysis.

Characteristic	Datasets, *n*	Samples, *n*
All data	18	247
Defined medium	9	126
Maize	5	65
Peanut	4	56
NRRL 3357	–	63
AF13	–	59
CA14	–	52
Mixed isolates	–	73
Datasets with matched AFB1 measurements	14	54 paired observations *^a^*
Time-series annotation	7	–

*^a^* The 54 paired observations are the complete ABAS–AFB1 records in the validation table.

**Table 2:** Principal quantitative associations from the cross-dataset mixed-effects analysis.

Analysis	Sample Size	Estimate (95% CI)
ABAS–AFB1 Pearson correlation	54	r=0.734; $p=2.70\times 10^{-10}$
Linear OSAS term	247	0.700 (0.610–0.790); p<0.001
Quadratic OSAS term	247	−0.434 (−0.502 to −0.366); p<0.001
OSAS turning point	247 *^a^*	0.81 SD (0.67–0.95); predicted ABAS 0.366 (0.186–0.546)
Random-effects OSAS summary	18 datasets	0.289 (0.241–0.336); $I^{2}=26.2\%$
OSAS × LMRS mixed-effects model	235	0.284 (0.198–0.370); p<0.001
Random-effects interaction summary	18 datasets	0.284 (0.245–0.324); $I^{2}=26.7\%$
High OSAS/high LMRS adjusted ABAS	95	0.789 (0.661–0.917)
Matrix heterogeneity	235	p=0.71
Leave-one-dataset-out interaction	18 refits	Range 0.278–0.299

*^a^* The turning point and corresponding predicted ABAS were estimated from the fitted quadratic mixed-effects model. Dataset-specific summaries were combined using random-effects meta-analysis; interaction quadrant values are model-adjusted marginal means.

**Table 3:** Integrated evidence profile of candidate convergence nodes.

Gene	Functional Class	Meta-log_2_FC	ABAS *r*	Integrated Score (0–10)
*laeA*	Global regulator	0.76	0.62	8.5
*veA*	Velvet regulator	0.72	0.60	8.5
*aflR*	Cluster regulator	1.10	0.82	10
*sntB*	Stress regulator	0.82	0.61	8.5
*catC*	Antioxidant	0.69	0.57	8.0
*pex11*	Peroxisome	0.73	0.59	7.0
*foxA*	β-oxidation	0.78	0.63	7.5
*atfB*	Stress transcription factor	0.62	0.52	6.5
*ole1*	Desaturation	0.71	0.58	7.0
*acsA*	Acetyl-CoA metabolism	0.66	0.56	6.0
*ppoA*	Oxylipin metabolism	0.59	0.47	4.5

The score allocates 0–2 points each to meta-log_2_ fold change, ABAS correlation, and *k*ME; 0–1 point to motif/ChIP evidence and food-matrix support; and 0–2 points to perturbation support. Thresholds and evidence sources are provided in the supplementary source-data table. The *aflR* association is partly non-independent because *aflR* contributes to ABAS.

## Data Availability

All transcriptomic data analyzed in this study were obtained from publicly accessible repositories. The source studies were deposited in the NCBI Sequence Read Archive, Gene Expression Omnibus, BioProject, and USDA Ag Data Commons. The corresponding repository identifiers were SRP034649, PRJNA678857, GSE101899, GSE40202, SRP057550, USDA Ag Data Commons 25301068, USDA Ag Data Commons 25080164, and PRJNA238212. The 18 analytical datasets used in this study were defined from distinct experimental contrasts, strains, treatments, food matrices, or sampling conditions within these public source studies and were assigned the internal identifiers DS01–DS18 for concise presentation. Supplementary Table S1 provides the public source-study accessions and characteristics, and Supplementary Table S2 summarizes the analytical datasets. No new sequencing data were generated. The supplementary package provides harmonized source metadata, matched ABAS–AFB1 validation records, model summaries, candidate-prioritization results, and source tables for the figures and tables. The retained release does not include the original gene-level module-membership map, a complete sample-level SRC01–SRC08 mapping, or raw sample-level OSAS/LMRS vectors; these missing elements limit source-study leave-one-out, module-removal sensitivity, and observed-score distribution analyses. The remaining source-data tables are author-compiled, harmonized derivative tables based on the cited public records.

## Supplementary Materials

The following supporting information can be downloaded at: https://www.mdpi.com/article/10.3390/metabo16090652/s1, Supplementary Table S1 contains public source-study characteristics and accession information; Supplementary Table S2 contains analytical-dataset summaries; and Supplementary Data S1 contains harmonized source data and model outputs.

## Abbreviations

The following abbreviations are used in this manuscript:

ABAS	aflatoxin biosynthetic activity score
AFB1	aflatoxin B1
LMRS	lipid metabolic reprogramming score
OSAS	oxidative-stress adaptation score
